# Global motion filtered nonlinear mutual information analysis: Enhancing dynamic portfolio strategies

**DOI:** 10.1371/journal.pone.0303707

**Published:** 2024-07-11

**Authors:** Wenyan Peng, Mingkai Wen, Xiongfei Jiang, Yan Li, Tingting Chen, Bo Zheng

**Affiliations:** 1 School of Physics, Zhejiang University, Hangzhou, China; 2 College of Finance and Information, Ningbo University of Finance and Economics, Ningbo, China; 3 Department of Finance, Zhejiang Gongshang University, Hangzhou, China; 4 Department of Finance, Zhejiang University of Finance and Economics, Hangzhou, China; 5 School of Physics and Astronomy, Yunnan University, Kunming, China; 6 Collaborative Innovation Center of Advanced Microstructures, Nanjing University, Nanjing, China; Shandong Normal University, CHINA

## Abstract

The complex financial networks, with their nonlinear nature, often exhibit considerable noises, inhibiting the analysis of the market dynamics and portfolio optimization. Existing studies mainly focus on the application of the global motion filtering on the linear matrix to reduce the noise interference. To minimize the noise in complex financial networks and enhance timing strategies, we introduce an advanced methodology employing global motion filtering on nonlinear dynamic networks derived from mutual information. Subsequently, we construct investment portfolios, focusing on peripheral stocks in both the Chinese and American markets. We utilize the growth and decline patterns of the eigenvalue associated with the global motion to identify trends in collective market movement, revealing the distinctive portfolio performance during periods of reinforced and weakened collective movements and further enhancing the strategy performance. Notably, this is the first instance of applying global motion filtering to mutual information networks to construct an investment portfolio focused on peripheral stocks. The comparative analysis demonstrates that portfolios comprising peripheral stocks within global-motion-filtered mutual information networks exhibit higher Sharpe and Sortino ratios compared to those derived from global-motion-filtered Pearson correlation networks, as well as from full mutual information and Pearson correlation matrices. Moreover, the performance of our strategies proves robust across bearish markets, bullish markets, and turbulent market conditions. Beyond enhancing the portfolio optimization, our results provide significant potential implications for diverse research fields such as biological, atmospheric, and neural sciences.

## Introduction

The complex financial networks, integrating finance, economics, network science, and systems theory, are essential for quantifying the interactions and complexities within financial systems [1–5]. Analyzing interactions and correlations between financial entities enhances our understanding of the market dynamics, identifies systemic risks, and provides crucial insights into financial stability [6–10].

Recent studies have shown that topological structures in financial networks evolve over time [11–16]. Dynamic networks, which are more complex and noisy, are of more significant importance compared with static ones [17, 18]. The dynamic networks obtained from the matrix of Pearson correlation (PC) coefficients are pivotal in understanding the complex interactions within brain networks, particularly during cognitive tasks [19–22]. It is observed that the networks obtained from the matrix of nonlinear mutual information exhibit superior robustness compared to networks obtained from the matrix of PC coefficients [23–25]. Literature on dynamic nonlinear networks primarily focuses on the basic properties of nonlinear networks [26, 27], with relatively fewer applications in portfolio optimization compared to static networks, mainly due to their susceptibility to noises.

Portfolio optimization in finance is the optimal allocation of financial assets in different stocks [28, 29], mutual funds, bonds, digital currencies [16, 30, 31], etc. to maximize the returns with risk tolerance [32]. Recently, financial scholars have been delving into the intricate relationships and distinct differences between various financial markets [16, 33, 34]. A sophisticated portfolio optimization strategy includes various elements, but stock selection, asset allocation, and market timing are fundamental components [35, 36]. The fundamental theory of portfolio optimization, originating from the Markowitz framework [37, 38], bases investment allocation on the mean-variance analysis [31]. This theory necessitates selecting a specific set of stocks [39, 40], as it focuses on allocating proportions within a selected stock group. A diverse range of alternative methodologies, such as neural networks [41, 42], genetic algorithms [43], and hierarchical clustering [10, 44, 45], have been introduced to the portfolio optimization. Recent studies indicate that green cryptocurrencies offer diversification benefits [30, 31], with a growing body of research incorporating cryptocurrencies into investment portfolios [46, 47].

Among these methods, using the hierarchical clustering in the network topology has emerged as an efficient approach for selecting stocks to generate optimal portfolios [10, 28, 45, 48]. Researchers utilize diverse linear correlation methodologies across various financial markets to evaluate the investment potential of assets possessing unique topological features [10, 28, 49–52], and implement minimum variance correlation strategies to optimize portfolio weights [31, 46]. Nevertheless, the exploration of portfolio optimization through nonlinear network topologies remains limited.

The nodes of the minimum risk portfolio are always located on the outer leaves of the MST tree generated by using the hierarchical clustering [48, 53]. Traditional hierarchical clustering portfolios predominantly concentrate on stocks at the extremities or at the core of the market spectrum [10, 45, 52]. Our methodology, however, adopts a holistic approach by incorporating an analysis of the full sample of stocks. A common approach for constructing dynamic financial networks involves generating network graphs over moving windows for the portfolio selection in each period [10, 45]. However, if the moving windows duration approximates the number of nodes, statistical uncertainty increases, and the dynamic network becomes predominantly noise-influenced [12, 54, 55].

The global motion, extracted with random matrix theory (RMT), drives price movements in complex financial systems, underscores the inter-connectedness of global financial markets [56–58], and minimizes noises [52]. The RMT highly effective in large dimensional systems like stock markets, is filtering noises from financial time series [59–62]. Notably, several large eigenvalues in both the mutual information (MI) and PC matrices significantly deviate from the upper boundary of the eigenvalue distribution as predicted by the Wishart matrix [63]. The RMT is also effective for nonlinear matrices like MI, characterized by independent random elements drawn from a probability distribution [59, 63]. Previous findings indicate that only the large eigenvalues, which significantly deviate from random ones, contain substantial information about the network structure [64, 65] and contribute to the variability of the dynamic system [66, 67]. Studies pertaining to the nonlinear RMT are notably limited, with its application within financial markets being almost entirely absent. Thus, we can extract the global motion matrix determined by the largest eigenvalue [52]. However, the structure and function of networks generated by the global motion of nonlinear matrices have not been sufficiently investigated.

The MI metric, rooted in the Shannon entropy theory, excels in evaluating nonlinear relationships, outperforming the PC, which is limited to linear associations [23]. The MI-based methodologies have been instrumental in constructing biological networks [68–71] and have recently gained prominence in complex systems and stock network studies [23, 72]. Research focusing on the dynamic structural characteristics of core and periphery nodes in stock networks has shown that peripherality serves as a reliable indicator for identifying optimal assets [45, 52]. It has been observed that peripheral stocks, when selected based on the MI, particularly with high frequency data, significantly outperform those chosen via PC [73]. What is more, the complex networks generated by the MI is still subject to noise interference, which can weaken the performance of investment portfolio constructed based on it.

In this study, our primary focus is on the global motion filtered MI, which could reduce noise and enhance timing strategies in dynamic stock networks. Initially, we construct dynamic stock networks with four matrices: MI, PC, and their corresponding global motion filtered matrices, based on daily returns of CSI 300 and S&P 500 component stocks. Then, we recognize peripheral stocks in these networks and build corresponding investment portfolios. Besides, we utilize eigenvalue growth and decline patterns associated with global motion to identify trends in collective market movement, exposing distinctive portfolio performance during periods of enhanced and weakened collective movements. In addition, we analyze Sharpe ratios across portfolios varying in stock numbers and holding duration, and unveil contrasting investment tendencies in the Chinese and American markets.

## Materials and methods

### Data description

Our study involves data on the constituent stocks of two major indices: the CSI 300 from the Chinese stock market and the S&P 500 from the American stock market. The dataset, spanning a decade (2009 to 2019), is freely accessible from the financial historical archives on https://cn.investing.com/. The collection and analysis methods adhered to the terms and conditions specified by the data source. In this analysis, the CSI 300 dataset encompass 2431 days, while the S&P 500 cover 2517 days. To ensure data continuity and integrity, stocks suspended for over 120 consecutive days are excluded. Consequently, the final dataset include 188 stocks from the CSI 300 and 420 from the S&P 500. The chosen dataset concludes in 2019, representing the most recent and comprehensive data unaffected by significant trade disruptions, including the COVID-19 pandemic.

### Construction of the mutual information and Pearson correlation networks

Initially, we compute correlation for component stocks of CSI 300 and S&P 500 market with moving windows. The closing price of the *x*th stock on day *t* is represented by *P*_*x*_(*t*), with the logarithmic return calculated as *R*_*x*_(*t*) = ln(*P*_*x*_(*t*)) − ln(*P*_*x*_(*t* − 1)). For each day *t*, the normalized return is computed within a moving time window set to Δ*T* = 125 days,

$$\begin{array}{c} r_{x}\left(t\right)=\left[R_{x}\left(t\right)-\left\langle R_{x}\left(t-t^{\prime }\right)\right\rangle \right]/\delta \left(t\right). \end{array}$$ (1)

In the expression where 〈…〉 denotes the time-averaged value over *t*′ in the past Δ*T* days, with *t*′ ranging from *t* − Δ*T* + 1 to *t*, and *δ*(*t*) is the standard deviation of the returns.

In the information theory, Shannon Entropy quantifies the uncertainty or unpredictability of a random variable or vector [73]. The normalized return *r*_*x*_(*t*) is uniformly divided into *N*_*x*_ sub-intervals, each with a width Δ*x* = (*x*_*max*_ − *x*_*min*_)/*N*_*x*_. The probability of stock *x* falling into the *i*th sub-interval is estimated by computing its occurrence frequency *f*(*x*_*i*_) within that interval. The density function is subsequently approximated by:

$$\begin{array}{c} p\left(x_{i}\right)\approx \frac{f(x_{i})}{\Delta T}. \end{array}$$ (2)

The entropy of a discrete random variable *r*_*x*_(*t*) is defined as:

$$\begin{array}{c} H\left(x\right)=-\sum_{i=1}^{N_{x}}p\left(x_{i}\right)log\left(p\left(x_{i}\right)\right). \end{array}$$ (3)

For discrete random variables *r*_*x*_(*t*) and *r*_*y*_(*t*), their joint entropy is defined as:

$$\begin{array}{c} H\left(x,y\right)=-\sum_{i=1}^{N_{x}}\sum_{j=1}^{N_{y}}p\left(x_{i},y_{i}\right)log\left(p\left(x_{i},y_{i}\right)\right), \end{array}$$ (4)

where *p*(*x*_*i*_, *y*_*i*_) represents the joint density function of variables X and Y. Herein, we employ *N*_*x*_ = 10 and 15 for calculating the mutual information, presenting results specifically for *N*_*x*_ = *N*_*y*_ = 10 since the results are highly similar.

The MI, which originates from the entropy information theory, measures a generalized, nonlinear relationship between two variables *r*_*x*_(*t*) and *r*_*y*_(*t*). It is defined as:

$$\begin{array}{c} I\left(x,y\right)=\sum_{i=1}^{N_{x}}\sum_{j=1}^{N_{y}}p\left(x_{i},y_{i}\right)log\frac{p(x_{i},y_{i})}{p\left(x_{i}\right)p\left(y_{i}\right)}. \end{array}$$ (5)

When *r*_*x*_(*t*) and *r*_*y*_(*t*) are independent, their joint density function satisfies *p*(*x*_*i*_, *y*_*i*_) = *p*(*x*_*i*_) ⋅ *p*(*y*_*i*_), generating a mutual information *I* of zero. From Eq 5, we derive that mutual information *I*(*x*, *y*) is expressed as *H*(*x*) + *H*(*y*) − *H*(*x*, *y*). Mutual information can be normalized to the interval [0, 1] [23], and is defined as:

$$\begin{array}{c} \text{NMI}\left(x,y\right)=\frac{2I(x,y)}{H(x)+H(y)}. \end{array}$$ (6)

The PC between the normalized return series of stocks x and y expressed as:

$$\begin{array}{c} C_{xy}\left(t\right)=\frac{\left\langle r_{x}\left(t\right)\cdot r_{y}\left(t\right)\right\rangle -\left\langle r_{x}\left(t\right)\right\rangle \cdot \left\langle r_{y}\left(t\right)\right\rangle }{\sqrt{\left(\left\langle r_{x}{\left(t\right)}^{2}\right\rangle -{\left\langle r_{x}\left(t\right)\right\rangle }^{2}\right)\cdot \left(\left\langle r_{y}{\left(t\right)}^{2}\right\rangle -{\left\langle r_{y}\left(t\right)\right\rangle }^{2}\right)}}. \end{array}$$ (7)

Within a network, the metric chosen defines the distance between nodes. The normalized distance based on MI between two stocks *x* and *y* is defined as:

$$\begin{array}{c} d\left(x,y\right)=1-\frac{I(x,y)}{H(x,y)}. \end{array}$$ (8)

Similarly, based on PC, the distance between stocks *x* and *y* is calculated using the following equation:

$$\begin{array}{c} d\left(x,y\right)=\sqrt{2(1-C_{x,y})}. \end{array}$$ (9)

### Extraction of the global motion in the MI and PC networks

Existing studies mainly focus on the application of the global motion filtering on the linear matrix to reduce the noise interference. Based on the above calculations, we get full correlation matrices (networks) named *C*_*ij*_ and *N*_*ij*_. Next, through global motion filtering, we apply global motion filtering to *C*_*ij*_ and *N*_*ij*_, i.e., $C_{ij}^{m}$ and $N_{ij}^{m}$. In the statistical physics, the statistical properties of eigenvalues are derived from the RMT matrix, formed from uncorrelated time series of finite length [57]. Here, the total number of stocks is denoted as *N*, and the aggregate data duration is represented as *T*. In the limit *N* → ∞ and *T* → ∞, maintaining *Q* ≡ *T*/*N* ≥ 1, the eigenvalue probability distribution *P*_*rm*_(λ) is described by the following expression:

$$\begin{array}{c} P_{rm}\left(\lambda \right)=\frac{Q}{2\pi }\frac{\sqrt{\left(\lambda_{+}-\lambda \right)\left(\lambda -\lambda_{-}\right)}}{\lambda }. \end{array}$$ (10)

The eigenvalues are constrained within the defined upper and lower bounds, given by $\lambda_{\pm }=[1\pm (1/\sqrt{Q}){]}^{2}$.

Expanding on the results of previous research [57], the correlation matrix *M*_*xy*_ is decomposed as:

$$\begin{array}{c} M_{xy}=\sum_{\alpha =1}^{N}\lambda_{\alpha }u_{x}^{\alpha }u_{y}^{\alpha }. \end{array}$$ (11)

The correlation matrix *M* encompasses both the PC matrix *C* and the MI matrix *N*. Here, λ_*α*_ represents the *α*-th eigenvalue of *M*_*xy*_, while $u_{x}^{\alpha }$ denotes the *x*-th component of the *α*-th eigenvector. Additionally, *N* signifies the total number of stocks under consideration.

In this manuscript, we focus on the global motion associated with the largest eigenvalue. The definition of the global correlation matrix is as follows:

$$\begin{array}{c} M_{xy}^{m}=\lambda_{m}u_{x}^{m}u_{y}^{m}, \end{array}$$ (12)

where λ_*m*_ denotes the largest eigenvalue of the matrix *M*, and $u_{x}^{m}$ is identified as the *x*-th component of the largest eigenvector. The matrix *M*^*m*^ is the result of noise reduction using the global motion approach, demonstrating, notable stability. Within this theoretical model, *N*^*m*^ corresponds to the global motion matrix constructed based on MI, and *C*^*m*^ represents the global motion matrix derived from PC.

### Calculation of the node peripherality in the MI and PC networks

We utilize the PMFG technique to construct sparse networks, employing both correlation networks *N*_*ij*_ and *C*_*ij*_, along with their corresponding global motion networks $N_{ij}^{m}$ and $C_{ij}^{m}$ for each day *t*. The PMFG approach, based on iterative creation of a constrained, planar graph, retains the most significant correlations among connected nodes, as elaborated in [74]. Following methodologies in [45, 52], we employ a composite peripherality measure to assess the peripherality of individual nodes within these networks. This peripherality *Cp* in the networks is computed as follows:

$$\begin{array}{c} \begin{array}{cc} Cp & =\left(DC^{w}+DC^{u}+BC^{w}+BC^{u}-4\right)/4\left(N-1\right)\\ & +\left(E^{w}+E^{u}+C^{w}+C^{u}+EC^{w}+EC^{u}-6\right)/6\left(N-1\right), \end{array} \end{array}$$ (13)

where, DC, BC, E, C and EC represent degree centrality, betweenness centrality, eccentricity, closeness, and eigenvector centrality, respectively. The superscripts *w* and *u* correspond to the weighted and unweighted sparse networks filtered with PMFG. Central nodes in the network typically have lower *Cp* values, while peripheral nodes tend to have higher *Cp* values. The peripherality *Cp* value for each stock is computed. Subsequently, the hierarchical clustering method is applied to divide all the stocks into 10 groups based on their *Cp* values, where group ‘1’ signifies central stocks and group ‘10’ represents peripheral stocks. In our analysis, the portfolio selection is based on the network structure in the previous time window and subsequently employed as a strategy for the following investment horizon.

### Construction of portfolios based on MI and PC networks

The Markowitz portfolio optimization theory is a fundamental concept in the field of modern finance, guiding optimal portfolio construction, weight allocation, and asset diversification [75]. It provides a structured method to determine the optimal asset weights within a portfolio, either by maximizing the portfolio’s return for given risk level or by minimizing the risk for a specific return. The Markowitz approach is formulated as follows:

$$\begin{array}{c} \begin{array}{cc} \text{min}\; & \lambda \omega^{T}\Omega \omega -\left(1-\lambda \right)\omega^{T}\mu ,\\ \text{s.t.}\; & \left\{ \begin{array}{c} r^{T}\omega =\mu \\ \sum_{x=1}^{N}w_{x}=1.\\ w_{x}\geq 0 \end{array}\right. \end{array} \end{array}$$ (14)

We analyze portfolios comprising the *k* most peripheral stocks, identified by the highest *Cp* values, and contrast them with portfolios of central stocks, characterized by the lowest *Cp* values.

In our model, *Ω* represents the covariance matrix of the assets within the portfolio. The methodology adopted in this study precludes the practice of short selling, mandating that the weights ***ω*** assigned to the assets must be strictly positive. The target return *μ* is predetermined; however, in order to mitigate the risk of overfitting, the specification of *μ* is deliberately omitted, with the emphasis being exclusively placed on the minimization of portfolio risk. For a parameter value of λ = 1, the portfolio strategy exhibits a propensity towards diversification, emphasizing the minimization of risk as opposed to the maximization of returns. In this context, the portfolio assessments are conducted utilizing both uniform and Markowitz optimization approaches for asset weighting.

For the each portfolio under consideration on day *t*, the return accrued over the holding period of *τ* days is meticulously tracked and defined as follows:

$$\begin{array}{c} r\left(k,t,\tau \right)=\sum_{x=1}^{k}\omega_{x}\cdot r_{x}\left(t,\tau \right), \end{array}$$ (15)

where $r_{x}^{s}\left(t,\tau \right)=\left(P_{x}\left(t+\tau \right)-P_{x}\left(t\right)\right)/P_{x}\left(t\right)$, with *P*_*x*_(*t*) is the price of the *x*-th stock among the selected most peripheral (or central) stocks on day *t*, and *P*_*x*_(*t* + *τ*) is the corresponding price on day *t* + *τ*. The term *τ* designates the holding period, which is within the range of *τ* ∈ [1, 125]. We assign uniform weights as $\omega_{x}=\frac{1}{k}$, and Markowitz weights are deduced from Eq (14). The annualized cumulative return, denoted as $r_{x}^{a}$, is defined by the formula (1 + *r*_*x*_(*t*, *τ*))^250/*τ*^ − 1, where 250 represents the typical number of trading days within a year. Subsequently, we define the annualized return of the portfolio:

$$\begin{array}{c} r^{a}\left(k,t,\tau \right)=\sum_{x=1}^{k}\omega_{x}\cdot r_{x}^{a}\left(t,\tau \right). \end{array}$$ (16)

The Sharpe ratio is chosen as a metric to evaluate the performance of the portfolio, defined as:

$$\begin{array}{c} Sp\left(k,\tau \right)=\bar{r}\left(k,\tau \right)/\sigma_{p}\left(k,\tau \right), \end{array}$$ (17)

where $\bar{r}\left(k,\tau \right)={\langle r\left(k,t,\tau \right)\rangle }_{t}$ signifies the mean return computed over all instances of time *t* within the full span of the time series, as well as the standard deviation *σ*_*p*_(*k*, *τ*). When the return of a portfolio, denoted as *r*(*t*, *τ*), is represented by *r*^*a*^(*t*, *τ*), the symbol *Sp* signifies the annualized Sharpe Ratio.

The Sortino ratio which is a variation of the Sharpe ratio, only takes in to account downside/negative volatility. It is assumed that the upside volatility is a bonus for investment, and should not be considered risky. Therefore, the total standard deviation in the Sharpe ratio is replaced by the downside deviation in the Sortino ratio [32, 76]:

$$\begin{array}{c} St\left(k,\tau \right)=\bar{r}\left(k,\tau \right)/\sigma_{d}\left(k,\tau \right), \end{array}$$ (18)

where *σ*_*d*_(*k*, *τ*) is the target downside deviation.

### Identification of collective movement trend with the global motion

The global motion is governed by the largest eigenvalue, and drives the collective price movements in the complex financial systems [56, 57]. Using a moving time window, we compute the correlation matrix and its largest eigenvalue, generating a time series of the largest eigenvalues as λ^*m*^(*t*).

The Granger causality test is applied to assess the causal relationship between this eigenvalue series and market indices in both Chinese and American markets. While the largest eigenvalue series of *N*_*ij*_ do not exhibit causality, the largest eigenvalue series of *C*_*ij*_ pass the causality test. Therefore, we utilize the largest eigenvalue series of *C*_*ij*_ as an indicator for market timing, defining market conditions accordingly:

$$\begin{array}{c} \left\{ \begin{array}{c} W:\lambda^{m}\left(t\right)<\lambda^{m}\left(t-125\right),\\ E:\lambda^{m}\left(t\right)>\lambda^{m}\left(t-125\right). \end{array}\right. \end{array}$$ (19)

On the day t, a value of λ^*m*^(*t*) exceeding λ^*m*^(*t* − 125), is interpreted as an indication of enhanced collective movements, marked by an ‘E’ superscript. In contrast, if λ^*m*^(*t*) is less than λ^*m*^(*t* − 125), signifying weakening collective movements, is denoted with a ‘W’ superscript. This notation is consistently applied in all figures throughout the article that include the ‘E’ and ‘W’ superscripts.

The mean return during the ‘E’ period is characterized as follows:

$$\begin{array}{c} \begin{array}{cc} & {\bar{r}}^{E}\left(\tau \right)={\left\langle r\left(k,t,\tau \right)\right\rangle }_{t,k},\\ & \text{s.t.}\lambda^{m}\left(t\right)>\lambda^{m}\left(t-125\right), \end{array} \end{array}$$ (20)

where 〈…〉 refers to averaging over the number of stocks: k, and time: t, where t should satisfy the enhancement conditions specified in the formula below. Conversely, the average rate of return during the ‘E’ period can be calculated as ${\bar{r}}^{L}\left(\tau \right)$.

The concept of conditional probability, denoted as $P_{w}^{E}\left(\tau \right)=\frac{P(r^{+},E)}{P(E)}$, is widely recognized. Here, *P*(*r*^+^, *E*) signifies the occurrence where the rate of return exceeds zero during the ‘E’ period, and *P*(*E*) pertains to the condition during the ‘E’ period. The corresponding expression for the win rate is expressed as:

$$\begin{array}{c} P_{w}^{E}\left(\tau \right)=\frac{n_{r^{+}\left(k,t,\tau \right)}^{E}}{n_{r^{+}\left(k,t,\tau \right)}^{E}+n_{r^{-}\left(k,t,\tau \right)}^{E}}, \end{array}$$ (21)

where $n_{r^{+}\left(k,t,\tau \right)}^{E}$ denotes the number of days with a positive return rate during the ‘E’ period. The Eq 16 is well-established that the return rate *r* is contingent upon three variables: *k*, *t*, and *τ*. The number of stocks, denoted as *k* = *N*/10 is categorized into either peripheral or central tiers.

### Summary of methods

Our methodology is structured into four main parts to ensure logical coherence:

1. **Construction of Stock Correlation Matrix Using Moving Windows**: Initially, we compute correlation for component stocks of CSI 300 and S&P 500 market in different time periods (moving windows from *t* − 125 to *t*), utilizing either PC or MI correlation matrix. Then, we get full correlate matrices (networks) named *C*_*ij*_(*t*) and *N*_*ij*_(*t*). Through global motion filtering, we get the global motion filtered matrices $N_{ij}^{m}\left(t\right)$ and $C_{ij}^{m}\left(t\right)$. Since the global motion is determined by the largest eigenvalue, which diverges the farthest from the random ones and captures a substantial share of the variability of dynamic system [52, 66, 67]. At this stage, the matrices are *N* × *N* correlation matrices (networks).
2. **Sparse Matrix Generation via PMFG**: Utilizing the Planar Maximally Filtered Graph (PMFG) technique, we retain significant connections to obtain sparse matrices (networks). This process involves filtering to preserve only the most crucial edges within the networks.
3. **The node Peripherality**: Sparse matrix calculated using Eq 13, the peripherality of each stock (node) within the network are available at each day. There are eight matrices (networks) across two markets: *C*_*ij*_, *N*_*ij*_, $C_{ij}^{m}$, and $N_{ij}^{m}$. The peripheral node exhibits less closely connected to other nodes in the network, which are less exposed to risk [45, 52]. From these matrices, we can derive metrics from various matrices to identify optimal assets.
4. **Clustering and Hierarchical portfolios**: Based on the peripherality values of all nodes on the same day, we categorize all stocks into ten tiers. Subsequently, we calculate the Sharpe and Sortino ratios for different holding periods (*τ*) and various market states to evaluate the investment portfolio performance of the networks.

## Results

### Comparison of MI and PC networks

The RMT is employed using Eq 12 to discern the global motion in *N*_*ij*_ and *C*_*ij*_ matrices for both CSI 300 and S&P 500 markets, resulting in $N_{ij}^{m}$ and $C_{ij}^{m}$, respectively. Subsequent references to *N*_*ij*_ pertain to the MI, as defined in Eq 6.

**Fig 1:**
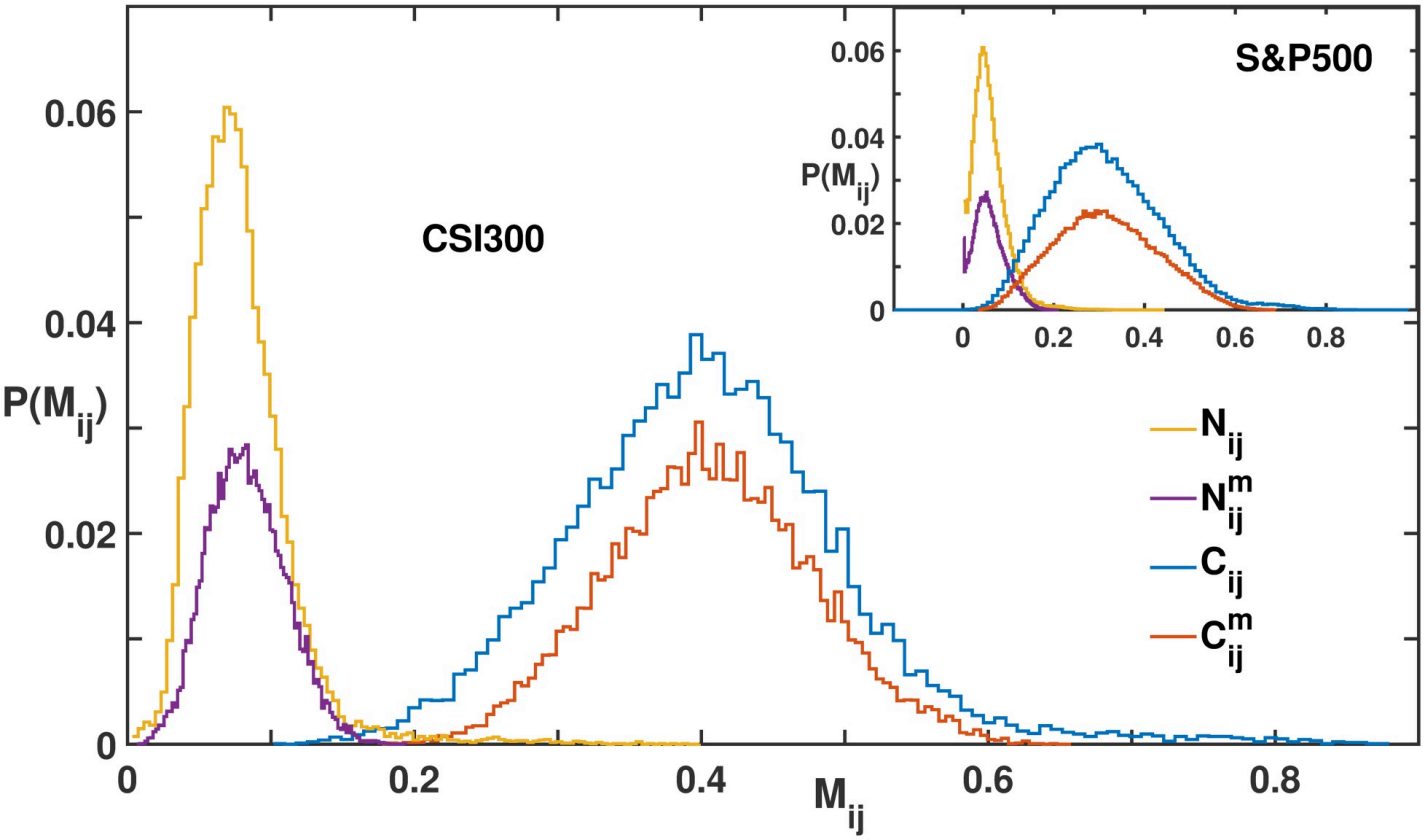
Probability distributions of the matrix elements *N*_*ij*_, $N_{ij}^{m}$, *C*_*ij*_ and $C_{ij}^{m}$, for the CSI 300 and S&P 500 markets.

Fig 1 presents the probability distributions of matrix elements for four matrices: *N*_*ij*_, $N_{ij}^{m}$, *C*_*ij*_ and $C_{ij}^{m}$. It is evident that *N*_*ij*_, similar to *C*_*ij*_, demonstrated normal distribution characteristics, aligning with typical Wishart matrices. The global motion matrices retain their original distributional characteristics. Both *N*_*ij*_ and *C*_*ij*_ preserve their symmetry axes, which is indicative of their respective market modes. Moreover, the figure highlights that in the CSI 300 market, the average values are around 0.08 for *N*_*ij*_ and 0.4 for *C*_*ij*_, whereas in the S&P 500, they are approximately 0.05 for *N*_*ij*_ and 0.32 for *C*_*ij*_, suggesting higher mean values in CSI 300 compared to S&P 500.

In our analytical assessment of the CSI 300 (with *L* = 2430, *N* = 188), it is observed that the eigenvalues λ_+_ and λ_−_ are 1.63 and 0.52, respectively. In contrast, for the S&P 500 (with *L* = 2516, *N* = 420) markets, these eigenvalues are determined to be λ_+_ = 1.98 and λ_−_ = 0.35. Several significant eigenvalues in the matrix notably deviate from the theoretical upper limit of the eigenvalue distribution characteristic of the Wishart matrix, typically representing the correlation matrix of uncorrelated time series [59, 63]. Eigenvalues confined within the interval [λ_−_, λ_+_] are classified as the random component. We quantify the degree of non-randomness in the matrix by calculating the ratio of eigenvalues exceeding this range to the total eigenvalue count, applied to both *N*_*ij*_ and *C*_*ij*_ matrices. The ratio is defined as follows:

$$\begin{array}{c} \begin{array}{c} ratio=n_{\lambda }/n, \end{array} \end{array}$$ (22)

where *n*_λ_ denotes the count of eigenvalues λ that are either less than λ_−_ or exceed λ_+_. As presented in Table 1, the ratio for the S&P 500 index is substantially higher compared to that of the CSI 300 index. Moreover, the ratio for *N*_*ij*_ is significantly greater than *C*_*ij*_. These observations imply that MI serves as a more robust measure of non-randomness than the PC coefficient when analyzing stock price data, which conclusion is consistent with the findings reported in Ref. [23].

**Table 1:** The eigenvalues beyond the range [λ_−_, λ_+_].

	CSI 300		S&P 500	
	*N*_*ij*_	*C*_*ij*_	*N*_*ij*_	*C*_*ij*_
*n*_λ_	182	53	411	242
ratio	0.968	0.282	0.979	0.576

*n*_λ_ is the number λ smaller than λ_−_ or larger than λ_+_, N for CSI 300 is 188, and N for S&P 500 is 420.

### Performance of the MI and PC network-based portfolios

Using the computed network peripherality *Cp* value for each stock based on Eq 13, we stratify *Cp* values in descending order and calculate the portfolio returns for each tier.

**Fig 2:**
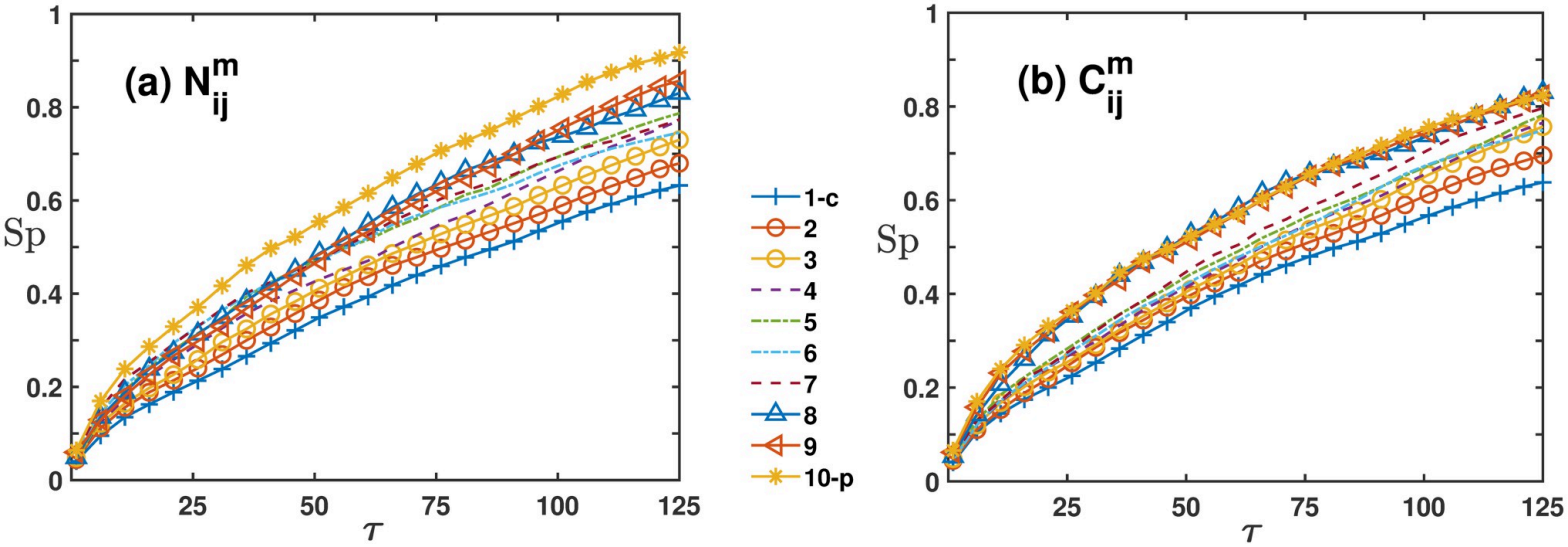
The comparison of Sharpe ratios in the S&P 500 global motion matrices for each tier. Sub-figure (a) shows the equal-weighted Sharpe ratio calculated through the $N_{ij}^{m}$ network, whereas sub-figure (b) is similar to (a) but displays the Sharpe ratio calculated through the $C_{ij}^{m}$ network.

Fig 2 shows the variation of the Sharpe ratios for $N_{ij}^{m}$ and $C_{ij}^{m}$ in the S&P 500 market over holding time *τ*, indicating that the Sharpe ratios for peripheral stocks are significantly higher than those of central stocks. The stratification and marginal Sharpe ratios of $N_{ij}^{m}$ are notably higher than those of $C_{ij}^{m}$.

In the Table 2, we display the average Sharpe ratio for each tier. the stocks within the first tier are identified as the most central in terms of their influence or connectivity, whereas those categorized in the tenth tier are recognized as the most peripheral, indicating their relatively lower significance or connectivity within the network.

**Table 2:** Sharpe ratios for each tier in the CSI 300 and S&P 500 markets.

	CSI 300				S&P 500			
Tier	*N*_*ij*_	$N_{ij}^{m}$	*C*_*ij*_	$C_{ij}^{m}$	*N*_*ij*_	$N_{ij}^{m}$	*C*_*ij*_	$C_{ij}^{m}$
1	0.122	0.098	0.137	0.125	0.424	0.384	0.418	0.399
2	0.157	0.130	0.164	0.147	0.434	0.418	0.431	0.431
3	0.182	0.152	0.183	0.156	0.448	0.447	0.456	0.455
4	0.184	0.181	0.182	0.153	0.474	0.472	0.474	0.464
5	0.193	0.211	0.203	0.174	0.506	0.502	0.489	0.480
6	0.195	0.231	0.188	0.218	0.543	0.497	0.512	0.469
7	0.204	0.236	0.203	0.224	0.525	0.508	0.547	0.493
8	0.207	0.215	0.214	0.229	0.535	0.527	0.549	0.548
9	0.224	0.200	0.198	0.239	0.520	0.523	0.535	0.548
10	0.219	**0.222**	0.208	0.193	0.492	**0.596**	0.482	0.555
c-half	0.1676	0.1544	0.1738	0.1510	0.4572	0.4446	0.4536	0.4458
per-half	0.2098	**0.2208**	0.2022	0.2206	0.5230	**0.5302**	0.5250	0.5226

1–10: each tier of *Cp*-value. ‘1’ represents the most central stocks. c-half: Sharpe ratio for half of the central stocks. p-half: Sharpe ratio for half of the peripheral stocks.

The Sharpe ratios in the S&P 500 market substantially exceed those in the CSI 300 market, likely due to its higher maturity level and a larger number of stocks per tier. In the CSI 300 market, the Sharpe ratios for the tenth tier concerning $N_{ij}^{m}$ and $C_{ij}^{m}$ do not significantly surpass those of the ninth tier. However, in the S&P 500 market, while the Sharpe ratios for *N*_*ij*_ and *C*_*ij*_ in the tenth tier are lower than those in the ninth, the global motion matrices show the tenth tier Sharpe ratios notably outperforming the ninth tier.

Additionally, we calculate averages for the top five and bottom five tiers. It is evident that in both the CSI 300 and S&P 500 markets, the Sharpe ratios of peripheral stocks are larger than those of central stocks. Moreover, peripheral stocks in global motion networks exceed their full correlation counterparts, and peripheral stocks within *N*_*ij*_ outperform those within *C*_*ij*_. The mean Sharpe ratio of the top half peripheral stocks in the $N_{ij}^{m}$ network is 5% and 1% higher than *N*_*ij*_*network* in CSI 300 and S&P 500 market respectively. Especially, in S&P 500 market, the mean Sharpe ratio of peripheral stocks in the tenth tier in the $N_{ij}^{m}$ network is 21% higher than *N*_*ij*_ network. The opposite trend is observed for central stocks.

**Fig 3:**
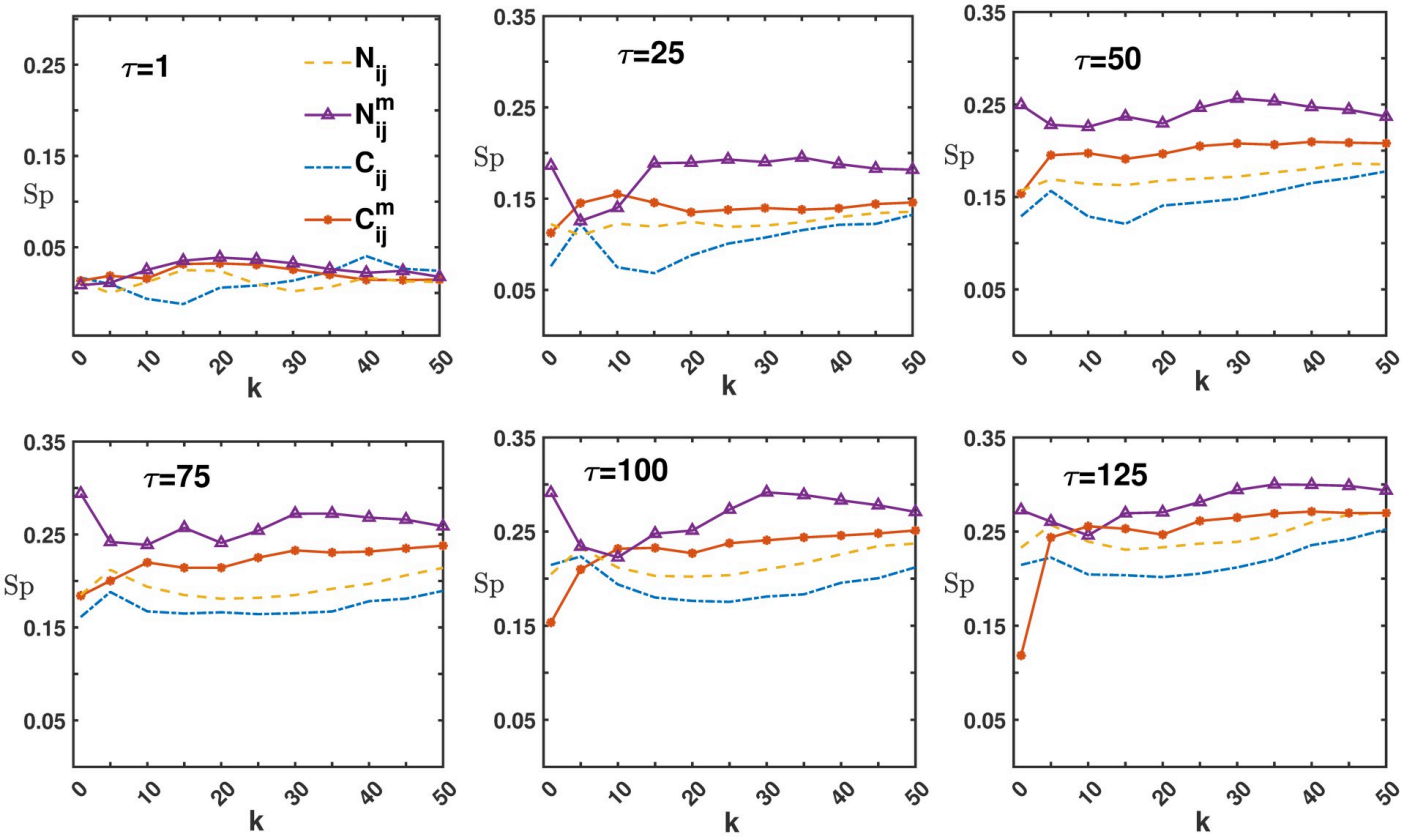
The Sharpe ratio difference of peripheral portfolios within *N*_*ij*_, $N_{ij}^{m}$, *C*_*ij*_ and $C_{ij}^{m}$ networks for the CSI 300 market in different holding times *τ* in the case of weights obtained by Markowitz optimization.

In Fig 3, we apply the Markowitz method to compare the Sharpe ratios of peripheral portfolios within four networks for the CSI 300, across holding periods *τ* = 1, 25, 50, 75, 100, 125. At *τ* = 1, differences in the Sharpe ratios of the peripheral portfolios across the four networks are minimal. This trend is more apparent for holding times from 25 to 125 days.

The results indicate that the Sharpe ratio is highest for $N_{ij}^{m}$, followed by $C_{ij}^{m}$, then *N*_*ij*_, and *C*_*ij*_ respectively. Notably, the peripheral portfolio in global motion filtered networks outperforms both *N*_*ij*_ and *C*_*ij*_, with *N*_*ij*_ exhibiting superior performance compared to *C*_*ij*_. The performance in the S&P 500 market mirrors the CSI 300 market findings, so further elaboration is omitted. In the following sections of this analysis, it is presupposed, unless explicitly stated otherwise, that the portfolio weights adhere to a uniform distribution.

### Portfolio performance under varying collective movement trends

**Fig 4:**
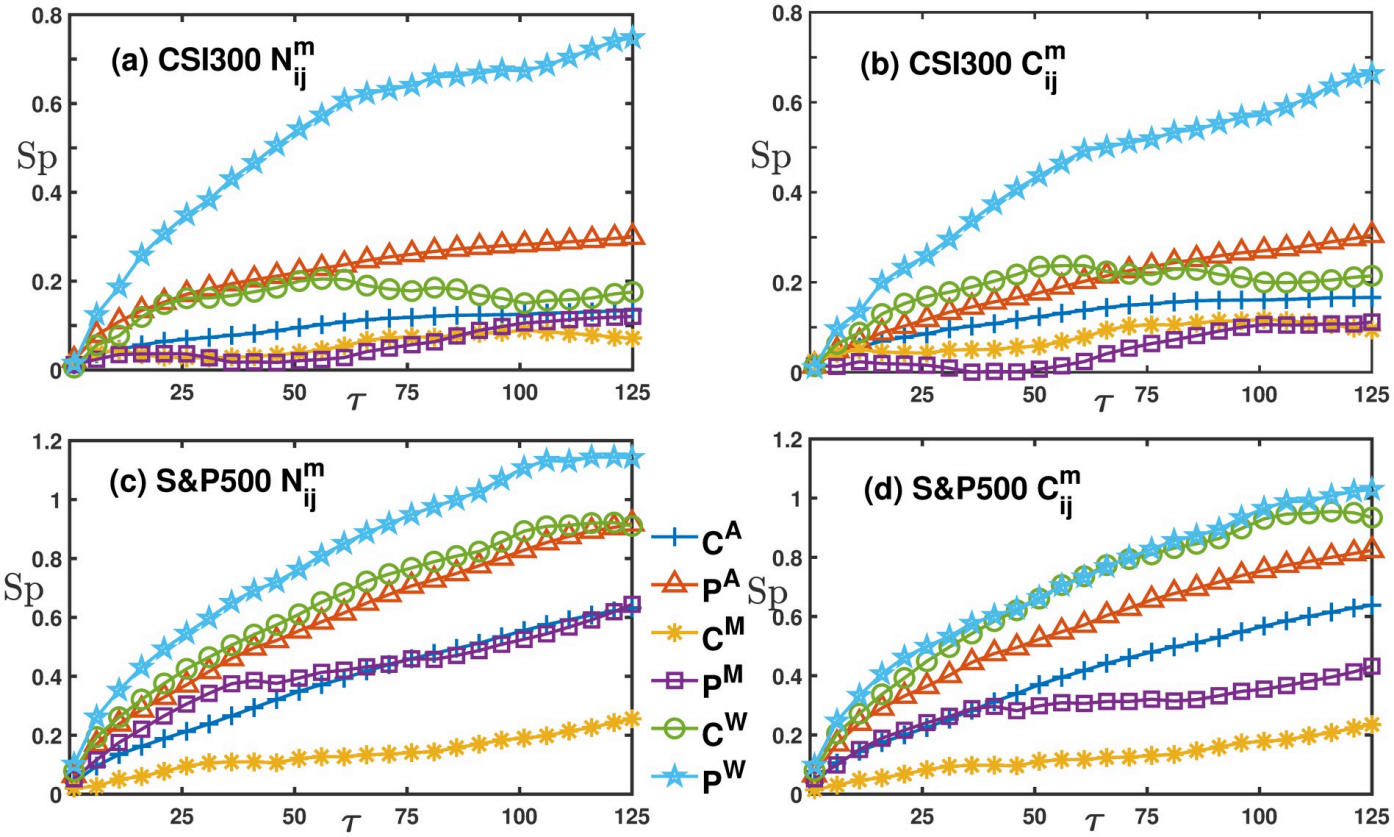
The comparison of Sharpe ratios of global motion filtered networks $N_{ij}^{m}$ and $C_{ij}^{m}$ in the CSI 300 and S&P 500 markets. Sub-figures (a) and (b) respectively represent the global motion networks of the CSI 300 market. Here, ‘C’ denotes the central node portfolio in the network, while ‘P’ represents peripheral nodes portfolio. The superscripts ‘E’ and ‘W’ indicate timing portfolios during collective movement strengthening and weakening, respectively, while those without superscripts refer to the entire time series. Sub-figures (c) and (d) are similar to (a) and (b), but they represent the S&P 500 market.

We employ the growth and decline patterns of eigenvalues corresponding to global motion to characterize trends in the collective movement of financial markets. The performance of portfolios during different market condition is assessed. We then calculate the Sharpe ratios of peripheral versus central nodes in the network during these distinct conditions, as depicted in Fig 4. For both CSI 300 and S&P 500 markets, peripheral nodes consistently exhibit the highest Sharpe ratios during ‘W’ market conditions, while central nodes had the lowest Sharpe ratios during ‘E’ market conditions. Importantly, regardless of whether the collective movements are strengthening or weakening, portfolios comprising peripheral nodes outperform those with central nodes in both $N_{ij}^{m}$ and $C_{ij}^{m}$ networks.

**Fig 5:**
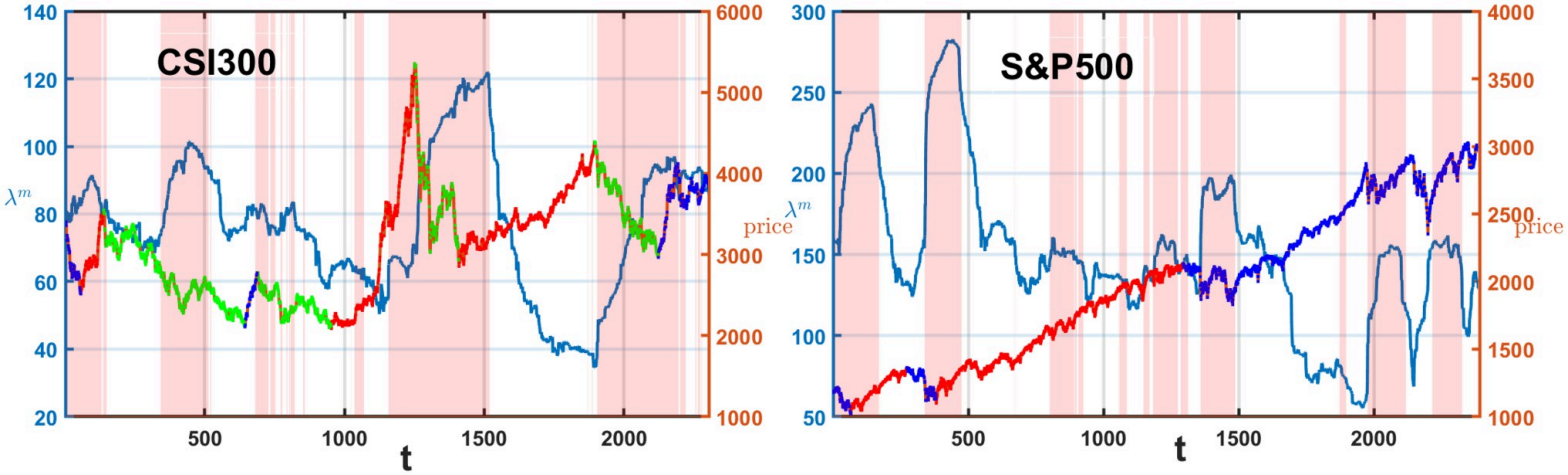
The largest eigenvalue corresponds to the price series of the index for sub-figure (a) CSI 300 and (b) S&P 500 market. The pink area represents the region of collective movement enhancement, while the remaining white area indicates the region of collective movement weakening. The green lines on the price chart mark the periods of bearish trends, the red lines highlight the bullish phases, blue lines are the periods of turbulence.

To understand the variables influencing the Sharpe ratio under different market conditions, we analyze the average return and win rate under conditions of ‘E’ and ‘W’. Fig 5 visualizes these conditions, with the pink area representing the region of ‘E’, and the white area representing ‘W’ period. In the CSI 300 market, ‘E’ comprises 1138 days, while ‘W’ spans 1167 days. For the S&P 500 market, ‘E’ cover 995 days, while ‘W’ extend over 1396 days. The orange line represents the corresponding index price. We modify Pagan and Sossounov’s (2003) method of dividing the market states into bullish, bearish, and range-bound markets [77, 78]. The peak is the highest price within an eight-month window before and after, and the trough is the lowest price in the same timeframe. To verify market trends, ensure there’s a confirmed uptrend or downtrend exceeding 20 percent change in value over a period longer than four months, starting from the identified peak or trough. The green lines on the price chart signify bearish market states, the red lines delineate bullish market periods, and the blue lines indicate the range-bound market states. The blue lines in Sub-figure (a) appear at the beginning and the end of the CSI 300 index price chart. The S&P 500 index does not feature any green lines, indicating the absence of bearish periods.

**Fig 6:**
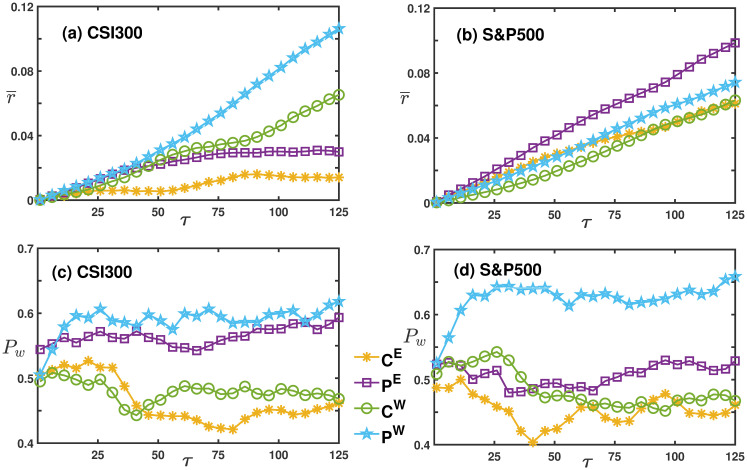
The average returns and win rates versus the holding period *τ* in the $N_{ij}^{m}$ network. Sub-figures (a) and (b) respectively show the average return for the CSI 300 and S&P 500, while sub-figures (c) and (d) represent the win rates. In these figures, blue pentagrams represent peripheral portfolios during the ‘W’ period, green circles represent central portfolios during the ‘W’ period, purple squares indicate peripheral portfolios during the ‘E’ period, and yellow snowflake shapes represent central portfolios during the ‘E’ period.

Fig 6 reveals that in the CSI 300 market (a and c), the peripheral portfolios have the largest average return and win rate during the ‘W’ period. This finding elucidates why the Sharpe ratio for the *P*^*W*^ line in Fig 4 is significantly larger than the others. In the S&P 500 market, although peripheral portfolios exhibit the largest average return during the ‘E’ period, their largest win rate is observed during the ‘W’ period. Consequently, the Sharpe ratio for *P*^*W*^ line is the largest in the S&P 500, but the difference between it and other Sharpe ratio curves is less marked compared to the CSI 300 market.

To examine the disparity in returns between peripheral portfolios and central portfolios across various time periods, we define *Pc* as the difference between the ratios (Sharpe ratios and Sortino ratios) of peripheral and central portfolios.

$$\begin{array}{c} Pc=<{Ratio}^{p}{>}_{k,\tau }-<{Ratio}^{c}{>}_{k,\tau }. \end{array}$$ (23)

We calculate the differential of Sharpe ratios and Sortino ratios between peripheral and central portfolios in Table 3. ‘A’ denotes the entire series data, ‘E’ corresponds to periods of enhanced collective movement, and ‘W’ signifies periods of weakened collective movement. Additionally, ‘U’ and ‘D’ indicate the timing of portfolios during bullish and bearish market states, respectively, while ‘T’ refers to periods of market turbulence. Firstly, by examining the columns, we observe that the values in columns $N_{ij}^{m}$ and $C_{ij}^{m}$ are greater than the non-global motion columns *N*_*ij*_ and *C*_*ij*_ for both the CSI 300 and S&P 500 markets. Furthermore, the values in columns $N_{ij}^{m}$ consistently exceed those in columns $C_{ij}^{m}$. Secondly, when examining the ‘Sp’ and ‘St’ rows within the CSI 300 market framework, we note significant discrepancies between peripheral and central portfolios during both the ‘W’ and ‘D’ periods. In contrast, the S&P 500 demonstrates a divergent behavior, with smaller differentials in the ‘E’ and ‘T’ periods compared to the more pronounced disparities observed in the ‘W’ and ‘U’ periods.

**Table 3:** Ratio of *Pc*.

		CSI 300				S&P 500			
		*N*_*ij*_	$N_{ij}^{m}$	*C*_*ij*_	$C_{ij}^{m}$	*N*_*ij*_	$N_{ij}^{m}$	*C*_*ij*_	$C_{ij}^{m}$
Sp	*Pc* ^*A*^	0.097	0.124	0.071	0.068	0.069	0.212	0.064	0.156
	*Pc* ^*E*^	-0.009	-0.000	-0.025	-0.030	0.062	**0.273**	0.054	0.169
	*Pc* ^*W*^	0.281	**0.366**	0.241	0.242	-0.005	0.163	0.006	0.033
	*Pc* ^*U*^	0.109	0.086	0.078	0.044	0.235	**0.393**	0.282	0.376
	*Pc* ^*D*/*T*^	0.272	**0.326**	0.244	0.245	-0.023	0.102	-0.038	0.025
St	*Pc* ^*A*^	0.083	0.145	0.045	0.059	0.110	0.323	0.121	0.225
	*Pc* ^*E*^	-0.193	0.022	-0.128	-0.034	0.066	**0.211**	0.055	0.138
	*Pc* ^*W*^	0.276	**0.277**	0.303	0.232	-0.001	0.139	0.050	0.052
	*Pc* ^*U*^	-0.075	-0.029	-0.069	-0.110	0.079	**0.251**	0.045	0.235
	*Pc* ^*D*/*T*^	0.228	**0.292**	0.214	0.220	-0.042	0.029	-0.045	-0.039

‘Sp’ is Sharpe ratio and ‘St’ is Sortino ratio. *Pc*: The ratios of peripheral tier portfolios minus the Sharpe ratio of central tier portfolios. The superscripts represent various time periods: ‘A’ denotes the entire series data, ‘E’ corresponds to periods of enhanced collective movement, and ‘W’ signifies periods of weakened collective movement. Additionally, ‘U’ and ‘D’ indicate the timing of portfolios during bullish and bearish market states, respectively, while ‘T’ refers to periods of turbulence market condition.

For the CSI 300, the most notable differential, 0.366, is observed under the $N_{ij}^{m}$ column and *Pc*^*W*^ row. In the S&P 500, the maximum differential of 0.273 appears under the $N_{ij}^{m}$ column and *Pc*^*E*^ row. In the bearish period ‘D’ within the CSI 300 market, the highest value recorded is 0.326. However, for the S&P 500 market during its bullish phase ‘U’, the largest value observed is 0.393. These variations are largely influenced by the trading habits of Chinese and American investors. In China, during periods of strengthening collective movement, the distinction between investing in peripheral or central stocks is minimal due to its strong synchronicity. However, during market differentiation, peripheral stocks in the Chinese market are more advantageous than central stocks. In the American market, during times of enhanced collective movement, peripheral stocks, unrestricted by price limits, tend to yield better returns than central stocks. Yet, when collective movement weakens, the returns on peripheral portfolios are comparable to those of central portfolios. Overall, the contrast between ‘E’ and ‘W’ periods is more pronounced in the Chinese market, while the difference in the American market is relatively subtle.

### Sharpe ratios heatmaps with different values of *k* and *τ*

The Sharpe ratio, as defined in Eq 18, is influenced by the number of stocks: *k*, and the holding period: *τ*. In this study, Sharpe ratios are computed for a heatmap of portfolio strategies, encompassing a range of 1 to 50 stocks and holding periods extending from 1 to 100 days. Each integral point along this continuum is representative of a distinct portfolio strategy. As illustrated in the supporting information, both the Markowitz optimization technique and equal-weighted portfolio strategies are utilized to generate Sharpe ratio heatmaps. Our analysis indicates that, in both the CSI 300 and S&P 500 markets, the Sharpe ratios derived from the equal-weighted and Markowitz methods exhibit minimal differences.

To determine the optimal number of stocks (*k*) and holding periods (*τ*) in portfolio strategies, we create annualized Sharpe ratio heatmaps for both the CSI 300 and S&P 500 markets.

**Fig 7:**
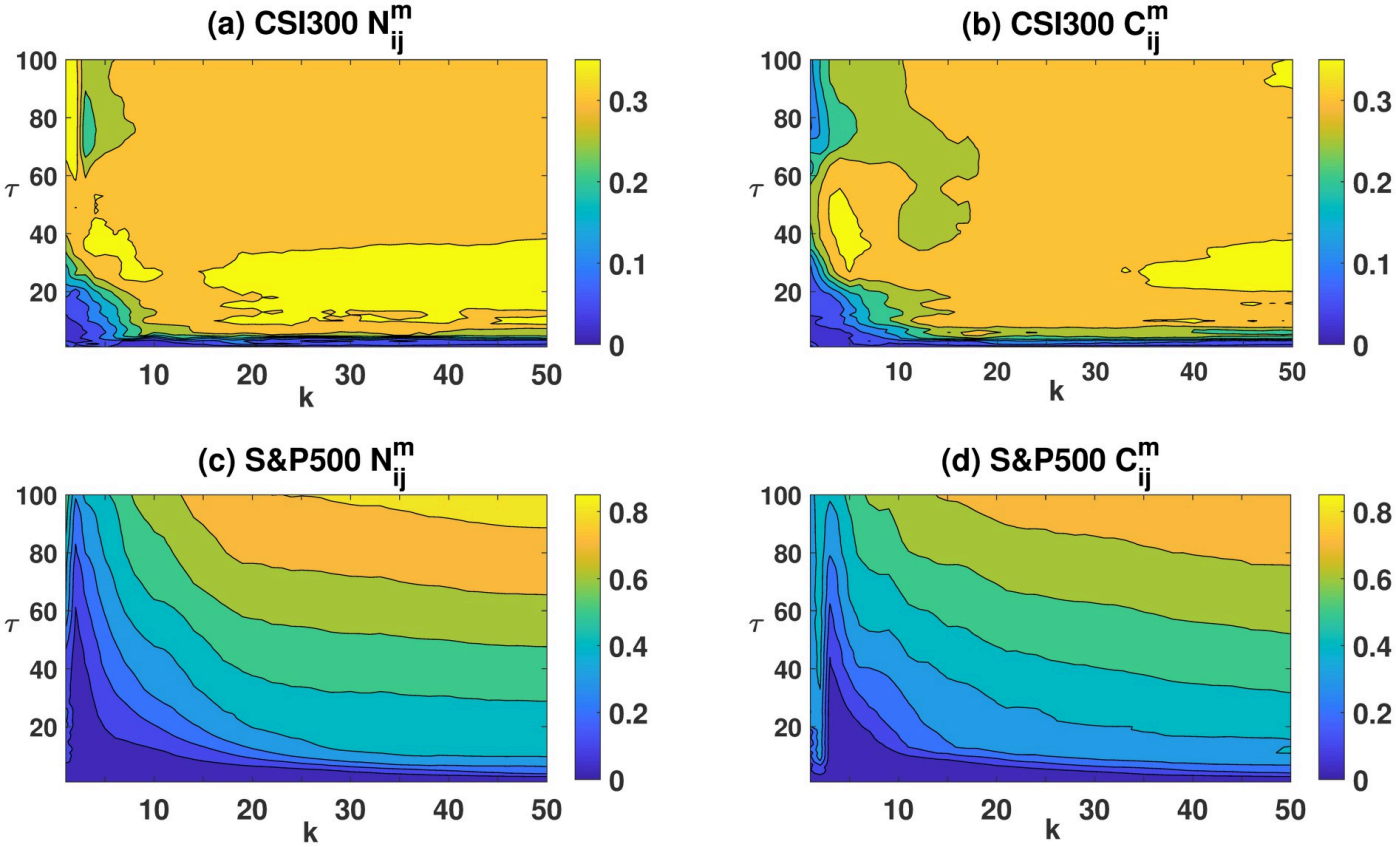
The annualized Sharpe ratio heatmaps of the CSI 300 and S&P 500 markets. Sub-figures (a) and (c) represent $C_{ij}^{m}$, while sub-figures (b) and (d) represent $N_{ij}^{m}$.

Fig 7 highlights the segments with the highest annualized Sharpe ratios. Specifically, sub-figures (a) and (b) delve into the dynamics within the CSI 300 market. In sub-figure (a), regions displaying the highest Sharpe ratios are mainly associated with portfolios comprising 40 to 50 stocks, and the holding period spans from 20 to 40 days. Conversely, sub-figure (b) emphasizes the highest Sharpe ratios in portfolios ranging from 20 to 50 stocks, with holding intervals spanning 10 to 30 days.

In contrast, the S&P 500 market demonstrates significantly higher annualized Sharpe ratios, especially apparent in the top-right quadrant of the figure. Although our computational limitations restrict our exploration into a broader range of stock counts and longer holding periods, the data available indicate that for the American market, the most effective investment strategy involves portfolios comprising more than 40 stocks and a holding period extending beyond 80 days, suggesting a long-term investment outlook. The CSI 300 market, however, seems to favor a shorter holding window of approximately 10 to 30 days, indicating a medium to short-term investment strategy. This finding is consistent with our expectations, as the Chinese stock market is relatively nascent, characterized by a significant proportion of retail investors who tend to pursue trend-following strategies. In contrast, the US stock market, with its more established history and a higher concentration of institutional investors, demonstrates a pronounced preference for risk diversification.

## Discussion and conclusion

Our paper introduces the global motion approach to filter nonlinear dynamic networks derived from the mutual information and first applies it to the timing analysis of dynamic investment portfolios. We utilize the daily price returns from constituents of the Chinese and American stock market indices, CSI 300 and S&P 500, to construct dynamic stock networks based on nonlinear matrices: MI and their corresponding global motion filtered matrices. We construct the linear PC matrices for comparison. The investment portfolios are constructed based on these networks. Our findings indicate that applying global motion to both MI and PC matrices effectively reduced noises in dynamic networks, thereby enhancing portfolio performance. Specifically, the portfolios at the periphery of global motion networks outperform both full networks based on MI and PC matrices. Notably, the Sharpe ratio of peripheral portfolio is the highest for global motion filtered MI networks. These findings imply that global motion can effectively filter noise in nonlinear networks, utilizing the network’s topological structure to identify optimal assets.

Numerous studies have constructed various timing indicators by analyzing the price and volatility of indices. However, pioneering the use of the strength of correlations among constituent stocks for timing is a novel approach. We employ the growth and decline patterns of eigenvalues of the global motion to characterize trends in the collective movement of financial markets. Our analysis reveals distinct portfolio performance during periods of enhanced and weakened collective movements. The optimal assets are frequently located at the peripheries of the global-motion-filtered MI network, especially during periods of weakened collective movement. We also divide the price series into three periods: bullish market, bearish market and turbulence conditions. Subsequently, we calculate the Sharpe and Sortino ratios for those respective periods. Our findings indicate that the difference between peripheral and central stocks is most pronounced in the bearish market for the Chinese market. Conversely, in the American market, this distinction is most significant during the bullish market periods. The performance of our strategies is robust across various market conditions. Moreover, the comparative analysis of Sharpe ratios among portfolios with different stock numbers and holding duration suggest a tendency toward short-term investments in the Chinese market and a preference for long-term investments in the American market. These observations underscore the significant advantages of integrating global motion in portfolio optimization strategies.

In conclusion, the Sharpe ratio of peripheral portfolios is the highest for global motion filtered MI networks, and the performance of the strategies is robust across various market conditions, indicating that applying global motion can effectively filter noise in nonlinear networks and enhance portfolio performance. Given the limitations of the linear correlation under extreme fluctuations, the nonlinear correlation demonstrates a broader applicability. The extensive application of nonlinear mutual information networks in various fields, such as biology, atmospheric sciences, and neural networks, the global motion-filtered mutual information likewise exhibits significant potential for application.

## Supporting information

S1 File Participant data. The data set supporting the findings of this study is available on Baidu Pan: https://pan.baidu.com/s/18yEPjhjvKN3hr9B8o6Ox9w?pwd=e56h (Access code: e56h).
(PDF)

S2 File Comparison of Sharpe ratios for different periods and Heatmaps of Sharpe ratios with equal-weighted and Markowitz optimization methods. The Supporting Information, S2 File, contains further analysis, some extra details about the methodology and some extra results.
(PDF)

S1 Data (ZIP)

## Abbreviations

MI: Multual information
PC: Pearson correlation
PMFG: Planar maximally filtered graph
RMT: Random matrix theory
