# Supplementary material for: Global motion filtered nonlinear mutual information analysis: Enhancing dynamic portfolio strategies
Source: PLoS One. 2024 Jul 11;19(7):e0303707. doi: 10.1371/journal.pone.0303707 (PMC11239051; doi:10.1371/journal.pone.0303707)
Supplement: S1 File — The data set supporting the findings of this study is available on Baidu Pan: https://pan.baidu.com/s/18yEPjhjvKN3hr9B8o6Ox9w?pwd=e56h (Access code: e56h). (PDF) [file pone.0303707.s001.pdf]

Procedures for Obtaining Data Used in Global Motion Filtered Nonlinear Mutual Information Analysis: Enhancing Dynamic Portfolio Strategies

Logging in to investing system.

Figure 1: Investing.com is a publicly accessible website that offers free access to global financial data. The available data includes a variety of financial instruments such as foreign exchange (forex), futures, indices, stocks, funds, and government bonds, as detailed in the following figure:

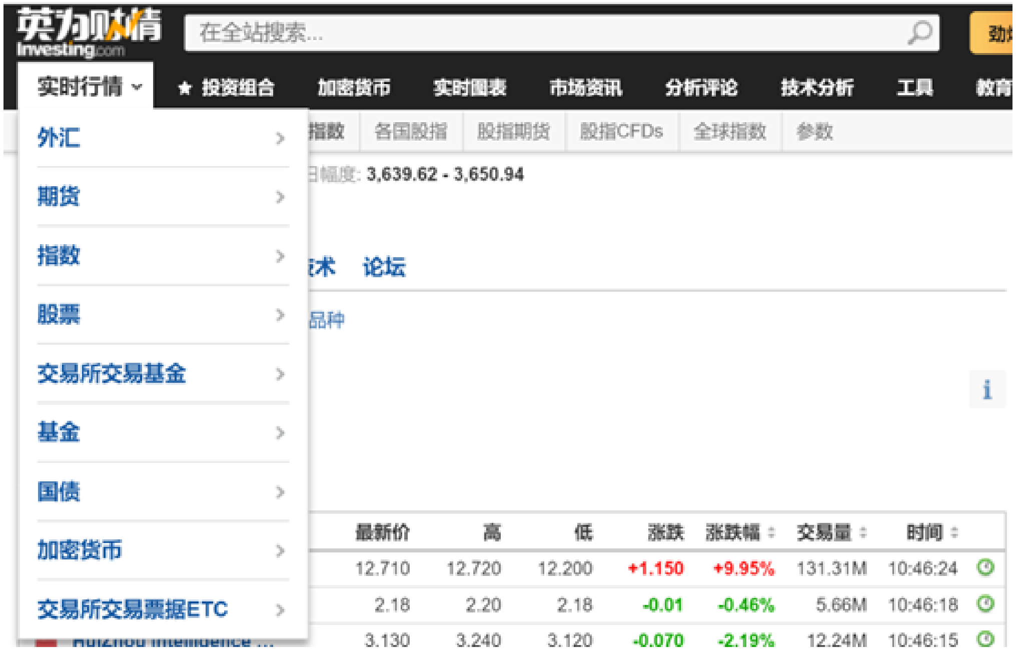

Figure 2: After accessing the website, we search for the S&P 500 index. Then, we find an option to select constituent stocks. By clicking on this option, we can view all the constituent stocks, as illustrated in the subsequent figure:

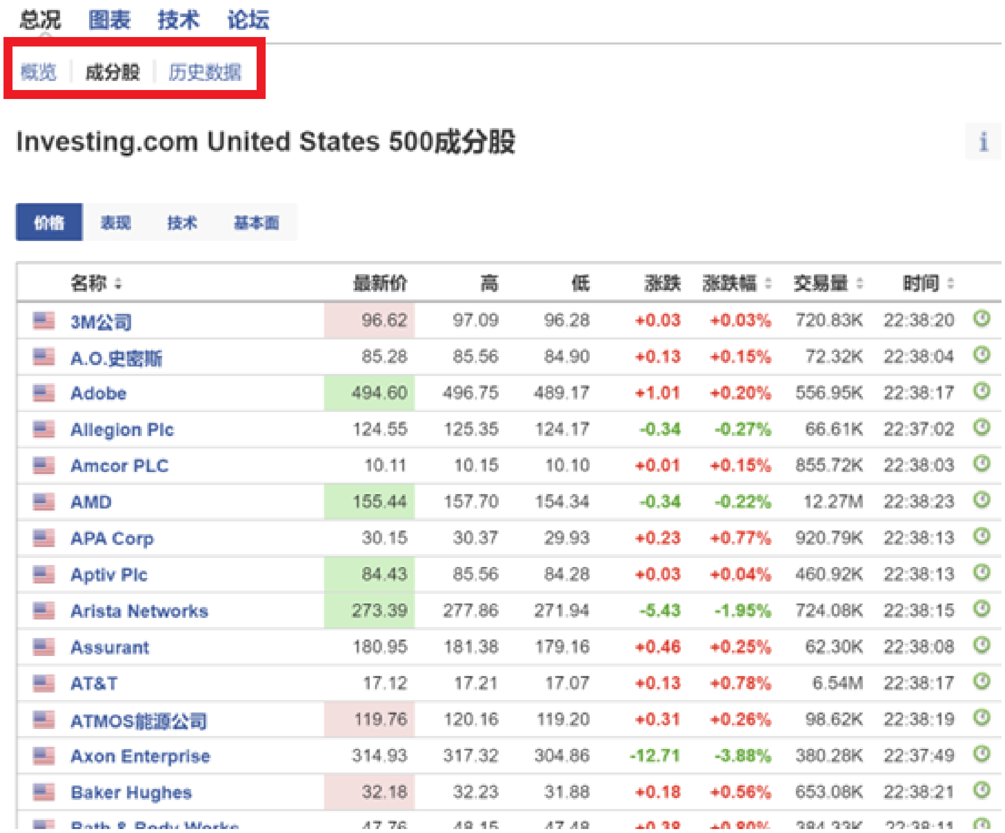

名称	最新价	高	低	涨跌	涨跌幅	交易量	时间
3M公司	96.62	97.09	96.28	+0.03	+0.03%	720.83K	22:38:20
A.O.史密斯	85.28	85.56	84.90	+0.13	+0.15%	72.32K	22:38:04
Adobe	494.60	496.75	489.17	+1.01	+0.20%	556.95K	22:38:17
Allegion Plc	124.55	125.35	124.17	-0.34	-0.27%	66.61K	22:37:02
Amcor PLC	10.11	10.15	10.10	+0.01	+0.15%	855.72K	22:38:03
AMD	155.44	157.70	154.34	-0.34	-0.22%	12.27M	22:38:23
APA Corp	30.15	30.37	29.93	+0.23	+0.77%	920.79K	22:38:13
Aptiv Plc	84.43	85.56	84.28	+0.03	+0.04%	460.92K	22:38:13
Arista Networks	273.39	277.86	271.94	-5.43	-1.95%	724.08K	22:38:15
Assurant	180.95	181.38	179.16	+0.46	+0.25%	62.30K	22:38:08
AT&T	17.12	17.21	17.07	+0.13	+0.78%	6.54M	22:38:17
ATMOS能源公司	119.76	120.16	119.20	+0.31	+0.26%	98.62K	22:38:19
Axon Enterprise	314.93	317.32	304.86	-12.71	-3.88%	380.28K	22:37:49
Baker Hughes	32.18	32.23	31.88	+0.18	+0.56%	653.08K	22:38:21
Bath & Body Works	47.76	48.15	47.48	+0.38	+0.80%	384.33K	22:38:11

Figure 3: We can select one of the constituent stocks and click through to its details.

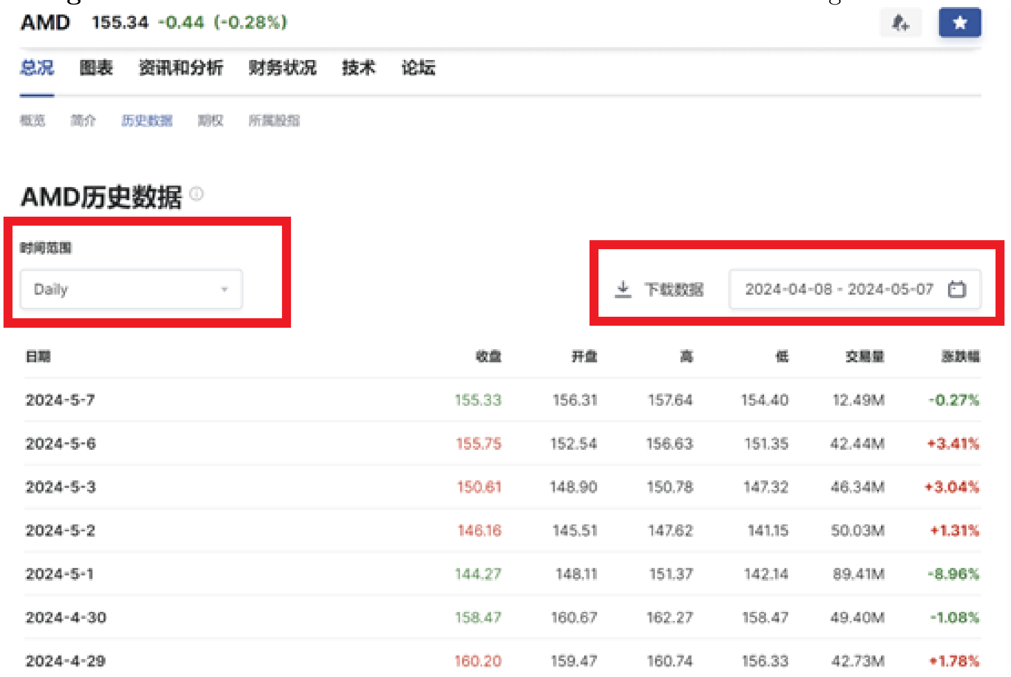

日期	收盘	开盘	高	低	交易量	涨跌幅
2024-5-7	155.33	156.31	157.64	154.40	12.49M	-0.27%
2024-5-6	155.75	152.54	156.63	151.35	42.44M	+3.41%
2024-5-3	150.61	148.90	150.78	147.32	46.34M	+3.04%
2024-5-2	146.16	145.51	147.62	141.15	50.03M	+1.31%
2024-5-1	144.27	148.11	151.37	142.14	89.41M	-8.96%
2024-4-30	158.47	160.67	162.27	158.47	49.40M	-1.08%
2024-4-29	160.20	159.47	160.74	156.33	42.73M	+1.78%

Figure 4: On the left side, outlined in red, we choose the frequency of data download, and on the right, we select the time period for the download. After selecting the desired data, we click the download button to save the information as a CSV file. The same method applies for downloading data for the CSI 300 constituent stocks.

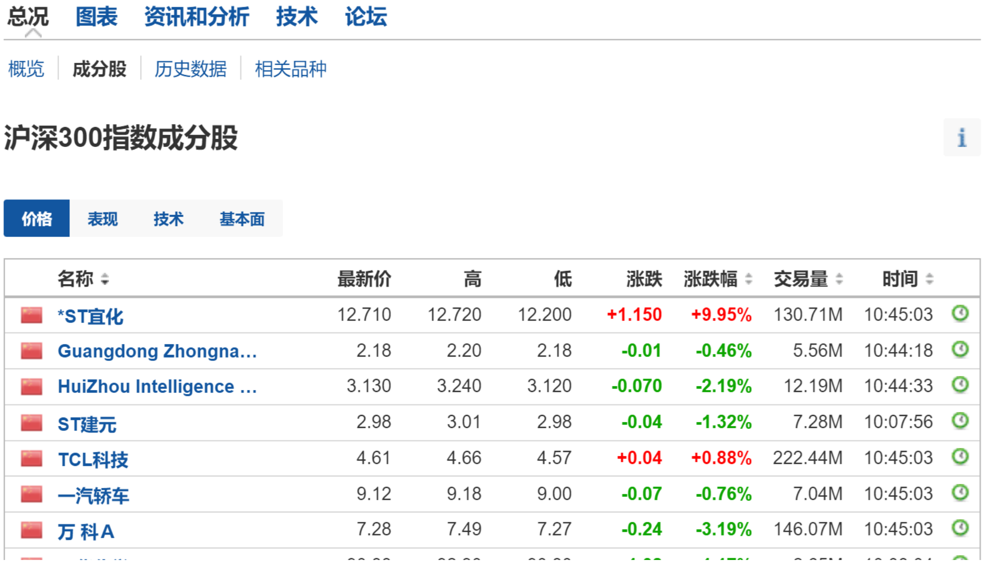
总况 图表 资讯和分析 技术 论坛

概览 | 成分股 | 历史数据 | 相关品种

沪深300指数成分股

价格 表现 技术 基本面

名称	最新价	高	低	涨跌	涨跌幅	交易量	时间
*ST宜化	12.710	12.720	12.200	+1.150	+9.95%	130.71M	10:45:03
Guangdong Zhongna...	2.18	2.20	2.18	-0.01	-0.46%	5.56M	10:44:18
HuiZhou Intelligence ...	3.130	3.240	3.120	-0.070	-2.19%	12.19M	10:44:33
ST建元	2.98	3.01	2.98	-0.04	-1.32%	7.28M	10:07:56
TCL科技	4.61	4.66	4.57	+0.04	+0.88%	222.44M	10:45:03
一汽轿车	9.12	9.18	9.00	-0.07	-0.76%	7.04M	10:45:03
万 科A	7.28	7.49	7.27	-0.24	-3.19%	146.07M	10:45:03
[illegible]	[illegible]	[illegible]	[illegible]	[illegible]	[illegible]	[illegible]	[illegible]

This method requires us to download data for each constituent stock individually. However, we can expedite the process by writing a web scraping script to quickly download the data for all constituent stocks.
