# Supplementary material for: Global motion filtered nonlinear mutual information analysis: Enhancing dynamic portfolio strategies
Source: PLoS One. 2024 Jul 11;19(7):e0303707. doi: 10.1371/journal.pone.0303707 (PMC11239051; doi:10.1371/journal.pone.0303707)
Supplement: S2 File — The Supporting Information, S2 File, contains further analysis, some extra details about the methodology and some extra results. (PDF) [file pone.0303707.s002.pdf]

Supporting information for: Global Motion Filtered Nonlinear Mutual Information Analysis: Enhancing Dynamic Portfolio Strategies

Wenyan Peng[1], Xiongfei Jiang[2], Mingkai Wen[2], Yan Li[4], Tingting Chen[3*], Bo Zheng[1,5,6*]

1 School of Physics, Zhejiang University, Hangzhou 310018, China
2 College of Finance and Information, Ningbo University of Finance and Economics, Ningbo 315175, China
3 Department of Finance, Zhejiang University of Finance and Economics, Hangzhou 310018, China
4 Department of Finance, Zhejiang Gongshang University, Hangzhou, 310018, China
5 School of Physics and Astronomy, Yunnan University, Kunming 650091, China
6 Collaborative Innovation Center of Advanced Microstructures, Nanjing University, Nanjing 210093, China

*ttchen@zufe.edu.cn; bozheng@zju.edu.cn

comparison of Sharpe ratios for different periods

Fig.1 shows the variation of Sharpe ratios for N_{ij}^m and C_{ij}^m in the S&P500 market over holding time τ, indicating that the Sharpe ratios for marginal (peripheral) stocks are significantly higher than those of central stocks in different periods. It can be observed that in the Chinese market, during 'D', bearish periods, the Sharpe ratio of peripheral stocks is significantly higher than that of central stocks, whereas in the U.S. market, during 'U' bullish states, the Sharpe ratio of peripheral stocks is noticeably higher than that of central stocks.

Heatmap of Sharpe ratios with equal-weighted and Markowitz optimization methods

As illustrated in Fig.2, both the Markowitz optimization technique and equal-weighted portfolio strategies were used to generate Sharpe ratio heatmaps. Our analysis indicates that, in both the CSI300 and S&P500 markets, the Sharpe ratios derived from the equal-weighted and Markowitz methods exhibit minimal differences. However, the heatmap based on the equal-weighted approach exhibits more distinct zones. Notably, in the S&P500 heatmap, as the stock count escalates, there is a marked decline in the demarcation line, which levels off beyond a portfolio of 20 stocks. This suggests that in the S&P500 market, an effective investment strategy should include more than 20 peripheral stocks. Conversely, the Chinese market heatmap displays a relatively consistent demarcation line without significant fluctuations.

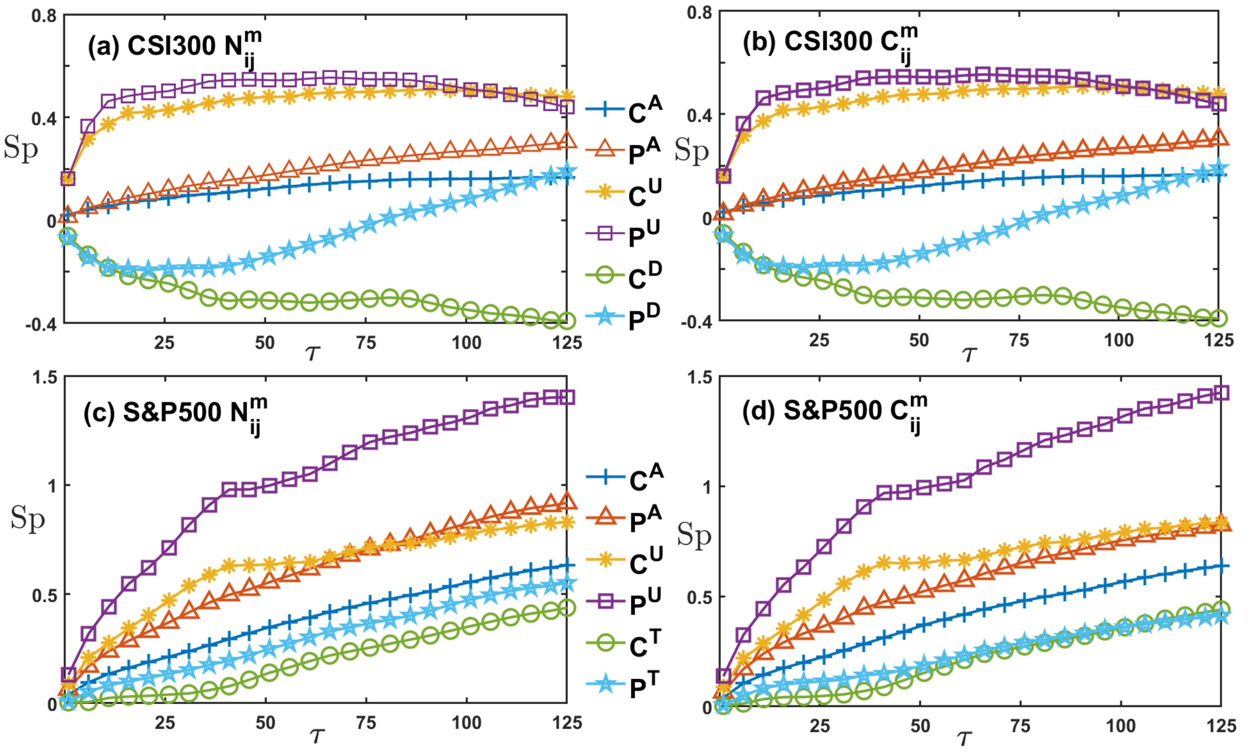

Fig 1. The comparison of Sharpe ratios of global motion filtered networks N_{ij}^m and C_{ij}^m in the CSI300 and S&P500 markets. Sub-figures (a) and (b) respectively represent the global motion networks of the CSI300 market. Here, 'C' denotes the central node portfolio in the network, while 'P' represents peripheral nodes portfolio. The superscripts 'U' and 'D' denote the timing of portfolios during bullish and bearish market states, respectively, while 'T' refers to periods of turbulence. The 'A' superscript represents the entire time series. Sub-figures (c) and (d) are similar to (a) and (b), but they represent the S&P500 market.

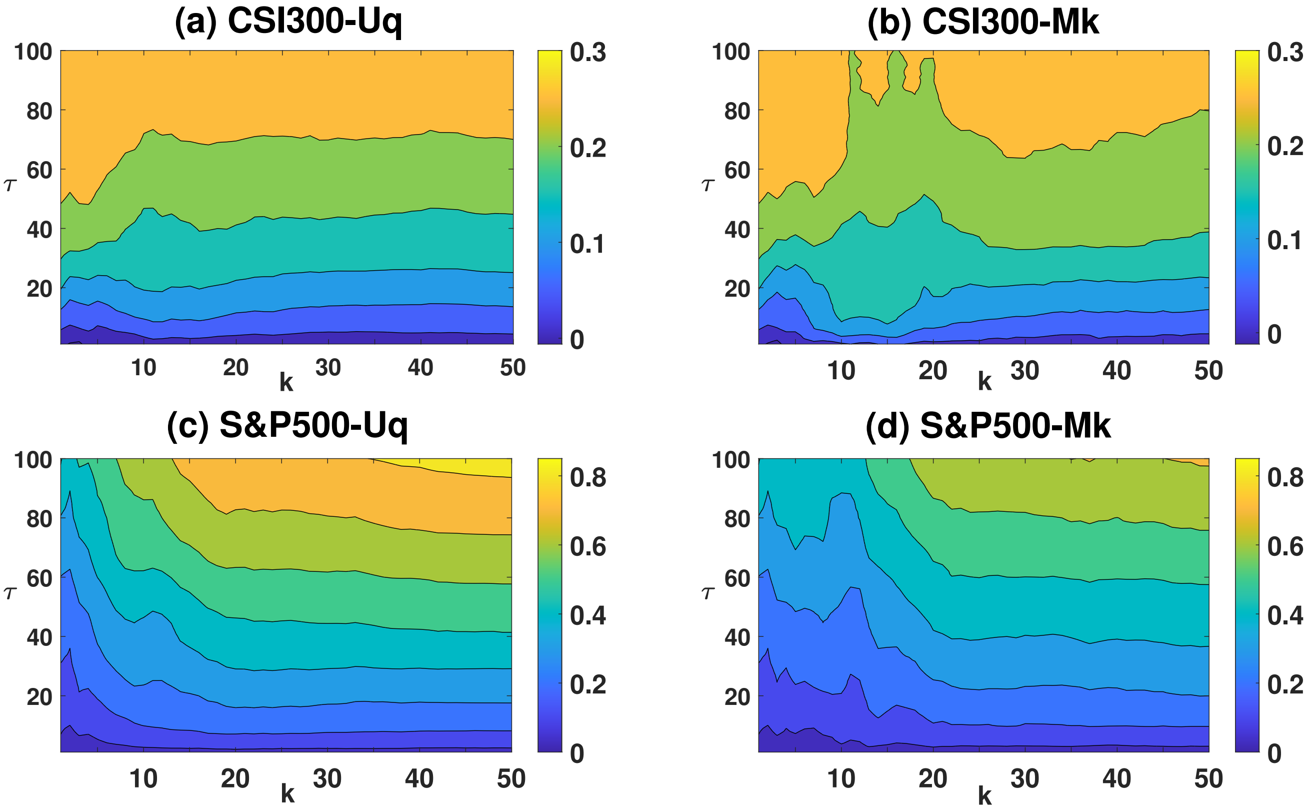

Fig 2. Heatmap of Sharpe ratios with equal-weighted and Markowitz optimization methods in N_{ij}^{m} networks. Sub-figures (a) and (c) are equal-weighted strategies, (b) and (d) are Markowitz optimization strategies

.
